# Cenp-meta is required for sustained spindle checkpoint

**DOI:** 10.1242/bio.20148490

**Published:** 2014-05-29

**Authors:** Thomas Rubin, Roger E. Karess, Zohra Rahmani

**Affiliations:** CNRS, Institut Jacques Monod, UMR7592, Université Paris Diderot, 75205 Paris Cedex 13, France; * Present address: Department of Genetics and Developmental Biology, Institut Curie, 75248 Paris Cedex 05, France.

**Keywords:** Mitosis, Spindle assembly checkpoint, Metaphase, Cenp-E, BubR1, APC/C, Kinetochore

## Abstract

Cenp-E is a kinesin-like motor protein required for efficient end-on attachment of kinetochores to the spindle microtubules. Cenp-E immunodepletion in *Xenopus* mitotic extracts results in the loss of mitotic arrest and massive chromosome missegregation, whereas its depletion in mammalian cells leads to chromosome segregation defects despite the presence of a functional spindle assembly checkpoint (SAC). Cenp-meta has previously been reported to be the *Drosophila* homolog of vertebrate Cenp-E. In this study, we show that *cenp-metaΔ* mutant neuroblasts arrest in mitosis when treated with colchicine. *cenp-metaΔ* mutant cells display a mitotic delay. Yet, despite the persistence of the two checkpoint proteins Mad2 and BubR1 on unattached kinetochores, these cells eventually enter anaphase and give rise to highly aneuploid daughter cells. Indeed, we find that *cenp-metaΔ* mutant cells display a slow but continuous degradation of cyclin B, which eventually triggers the mitotic exit observed. Thus, our data provide evidence for a role of Cenp-meta in sustaining the SAC response.

## INTRODUCTION

During normal mitosis, faithful chromosome segregation is assured by the robustness of kinetochore attachment to the spindle microtubules (K–MT attachments) and the surveillance mechanism called the spindle assembly checkpoint (SAC), which delays mitotic progression if incorrect K–MT attachments are detected, giving the cell time to correct them. The SAC functions to regulate temporally the activity of the anaphase promoting complex/cyclosome (APC/C), a ubiquitin ligase (Harper et al., 2002; Peters, 2002; Yu, 2002). By targeting Cyclin B and Securin for degradation by the proteasome, APC/C drives the cell to mitotic exit. Early in mitosis, several checkpoint proteins (including Mad1, Mad2, Bub1, BubR1, Bub3 and Mps1) bind to unattached or inappropriately attached kinetochores. This recruitment to the kinetochore generates a ‘stop anaphase’ signal that diffuses into the cytosol. This signal is composed of the checkpoint proteins Mad2, BubR1 and Bub3 bound to Cdc20, a key co-factor of the APC/C necessary for its activation (Musacchio and Salmon, 2007; Santaguida and Musacchio, 2009). Following correct attachment of all chromosomes to the spindle, the checkpoint is inactivated and Cdc20 is freed to activate the APC/C (Yu, 2002). Several additional factors have been shown to participate in mitotic checkpoint signalling in metazoans. These factors include the RZZ complex (Karess, 2005) and the protein CENP-E (Mao et al., 2003; Mao et al., 2005; Weaver et al., 2003).

Cenp-E is a plus-end directed molecular motor kinesin localized specifically to kinetochores during mitosis (Yen et al., 1991; Yen et al., 1992). Several studies have demonstrated that Cenp-E is required for efficient capture and attachment of microtubules to kinetochores (Lombillo et al., 1995; Wood et al., 1997; Yao et al., 1997; McEwen et al., 2001; Putkey et al., 2002; Kapoor et al., 2006). In mammals, Cenp-E has been shown to be required for chromosome congression to the spindle equator and for stable kinetochore–microtubule attachment. Depletion of Cenp-E by antisense oligonucleotides (Yao et al., 2000) or RNA interference (Tanudji et al., 2004) and inhibition of CENP-E recruitment to kinetochores by antibody microinjection (Schaar et al., 1997; McEwen et al., 2001) in human cells all lead to problems in chromosome congression. In primary mouse fibroblasts, CENP-E depletion results in chromosome segregation defects with a few chromosomes clustered around the spindle poles (Putkey et al., 2002). Similarly, depletion of Cenp-E by siRNA in HeLa cells causes chromosome missegregation with the presence of mono-oriented chromosomes localized close to the spindle pole (Tanudji et al., 2004). More recently, Cenp-E was shown to be phosphorylated *in vitro* and *in vivo* by both Aurora kinases A and B at a conserved site close to the CENP-E kinesin neck domain (Kim et al., 2010). This phosphorylation leads to reduced affinity for microtubules *in vitro* whereas preventing its phosphorylation leads to chromosome alignment defects, thereby demonstrating that Aurora kinases control Cenp-E mediated promotion of chromosome biorientation (Kim et al., 2010). Overall these combined observations indicate that CENP-E stabilizes K–MT attachment and promotes chromosome movement toward the metaphase plate.

Although it is now well established that CENP-E has a role in efficient K–MT capture and chromosome congression, its implication in the signaling cascade of the mitotic checkpoint is more controversial. Inhibition of CENP-E expression in mammalian cells, by antisense oligonucleotides or by RNAi, leads to prolonged mitotic arrest (Yao et al., 2000; Tanudji et al., 2004). On the other hand, depletion of CENP-E in primary mouse fibroblasts does not result in long-term mitotic arrest (Putkey et al., 2002). Similarly, in *Xenopus*, immunodepletion of Cenp-E from *Xenopus* extracts results in loss of mitotic arrest in the presence of microtubule-depolymerizing agents (Abrieu et al., 2000). Moreover, CENP-E and BubR1 can form a stoichiometric complex, and the presence of CENP-E can greatly enhance the kinase activity of BubR1 toward itself or an exogenous substrate such as histone H1 (Mao et al., 2003; Weaver et al., 2003; Guo et al., 2012). These observations suggest that the loss of mitotic arrest observed in *Xenopus* egg extracts depleted of CENP-E is caused by a reduction of BubR1 kinase activity.

In *Drosophila*, Cenp-meta and Cenp-ana were previously reported to be closely related to Cenp-E (Yucel et al., 2000). While a *cenp-ana* mutation leads to an increased frequency of anaphase, a *cenp-meta* mutation leads to an elevated prometaphase frequency (Yucel et al., 2000), reflecting a role in promoting chromosome congression, an observation confirmed by others (Williams et al., 2003; Maia et al., 2007). Moreover, co-depletion of *cenp-ana* and *cenp-meta* in *Drosophila* S2 cells by RNAi yield to a phenotype and a mitotic index identical to the single RNAi depletion of *cenp-meta* (Goshima and Vale, 2003) strongly suggesting that Cenp-meta is likely to be the *Drosophila* homolog of vertebrate Cenp-E. Zygotic deletion of Cenp-meta, is lethal at the pupal stage. Homozygous mutants of *cenp-meta* show an elevated mitotic index, with retarded congression of chromosomes to the metaphase plate (Yucel et al., 2000). Such a phenotype suggests that the checkpoint is functional and activated in these mutants, delaying anaphase onset since the chromosomes are not properly bioriented. Paradoxically, *cenp-metaΔ* mutants display a very high level of aneuploid cells (Yucel et al., 2000; Williams et al., 2003). This apparent discrepancy has not been further addressed until the present study. Here, we show that Cenp-meta is required for prolonged mitotic checkpoint maintenance.

## MATERIALS AND METHODS

### Genetic stocks

The strain containing the null allele mutation *cenp-metaΔ* has already been described previously. Briefly, imprecise excision of the P element deleted ∼5 kb of Cenp-meta genomic DNA just upstream of the 5′ end of the initial P element insertion site (Yucel et al., 2000). Flies expressing GFP-Rod, RFP-Rod, the mutations *mad2^P^* and *asp*, RFP-BubR1, GFP-Mad2, Spc25-mRFP1 transgene were described previously (Buffin et al., 2005; Buffin et al., 2007; Schittenhelm et al., 2007; Rahmani et al., 2009). GFP-cyclin B flies were a gift from J. Raff (Cancer Research UK Gurdon Institute, Cambridge, England, UK).

### Western blot

Protein extracts from 5 brains of wild-type and *cenp-metaΔ* homozygous third instar larvae were loaded onto SDS 8%-acrylamide gels. Proteins were transferred to nitrocellulose membrane (Protran BA 85; Schleicher and Schuell, Dassel, Germany) using a BioRad electrophoretic blotting device. Membranes were blocked for 1 hour in TBST (50 mM Tris-HCl, pH 7.4, 150 mM NaCl, 0.1% Tween 20) with 5% dry milk and incubated 1 hour 30 minutes at room temperature with rabbit anti-*Drosophila* Cenp-meta (a gift from M. L. Goldberg, Cornell University, Ithaca, NY) diluted 1:6000 in TBST plus 1% milk. After washing in TBST, the blot was incubated for 1 hour at room temperature with secondary antibody of goat anti-rabbit IgG conjugated with horseradish-peroxidase (Promega, Charbonnières, France) diluted 1:5000. Immunodetection was carried out with the SuperSignal Kit (Perbio Science France, Brebières, France).

### Cytology

Third instar larval brains were fixed and stained in aceto-orcein as previously described (Rahmani et al., 2009). The mitotic index (mean number of mitotic cells per microscopic field) in response to colchicine-induced depolymerization of microtubules was determined by preincubating brains in 10^−4^ M colchicine in 0.7% NaCl for 0, 30, or 60 minutes and then transferring them to 0.5% Na citrate hypotonic solution for 4 minutes before staining. Aneuploidy was determined by preincubating the brains in 10^−4^ M colchicine in 0.7% NaCl for 7 minutes to obtain a readable karyotype, then transferred to 0.5% Na citrate hypotonic solution for 4 minutes before being fixed and stained. Cells were observed with a microscope (Microphot; Nikon) and a 63× NA 1.4 phase contrast objective (Carl Zeiss, Inc.). A cell was reported as aneuploid if it clearly showed at least one extra chromosome.

### *In vivo* imaging

*In vivo* imaging of living neuroblasts of third instar larval brains were carried out as described previously (Rahmani et al., 2009). Brains were imaged in a temperature-controlled room set at 21°C with a spinning disk confocal head (Ultraview; PerkinElmer) mounted on an inverted microscope (DMI6000; Leica) with a Zeiss 100× NA 1.4 lens and a camera (QuantEM 512SC; Photometrics), all piloted by MetaMorph 7 (MDS Analytical Technologies). At 20-seconds intervals, a z series of images consisting of seven 1-µm steps was acquired with 1× binning. Confocal video frames are maximum intensity projections. Time-lapse image series were converted into videos with ImageJ software (National Institutes of Health), and still images were processed using Photoshop (Adobe). NEB was defined as when the RFP-Spc25 or RFP- or GFP-Rod signal began to be visible on kinetochores. Anaphase onset was defined as the moment sister kinetochores (marked with either Spc25 or Rod) began to separate. GFP-cyclin B degradation dynamics was measured as described previously (Rahmani et al., 2009). Briefly, neuroblasts expressing one copy of GFP-cyclin B and RFP-Rod were filmed as above. GFP fluorescence in each z section was quantified for the whole cell and for a central region containing the kinetochores and most of the spindle. This gave a more robust measurement of the OCBD because spindle-associated cyclin B is the first to be degraded during metaphase (Buffin et al., 2007). The signal was adjusted for background and for bleaching relative to the signal of a neighboring non-mitotic cell (assumed to be constant). In the graphs for Fig. 4, the signal levels for the whole cell are displayed as normalized signal relative to the maximal intensity measured for the cell.

### Statistical analysis

Data were expressed as mean ± s.d. The *P*-values were calculated using Student's *t*-test. Values were considered statistically different whenever *P*<0.05.

## RESULTS

### *cenp-metaΔ* mutant cells are checkpoint competent

**Fig. 1.**
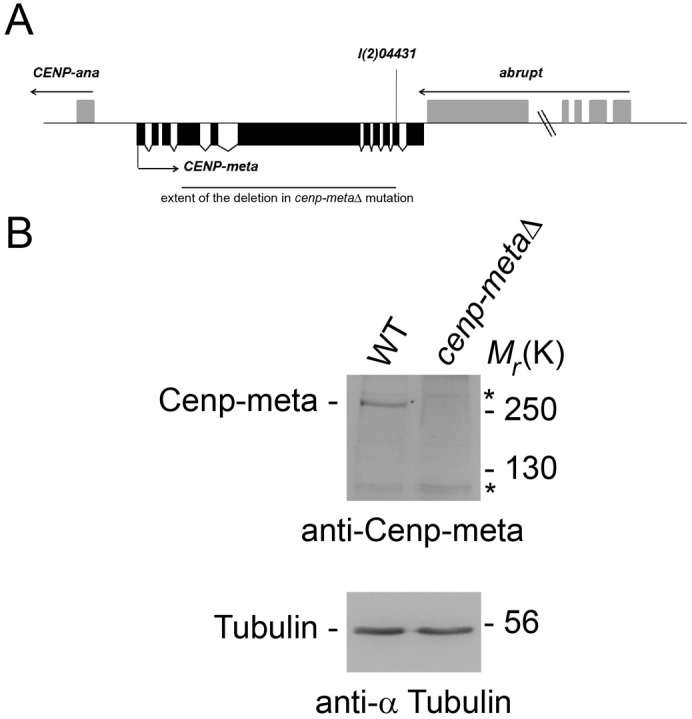
The *cenp-metaΔ* gene and gene product. (A) The *Cenp-meta* gene locus contains 11 exons and shares 55 nucleotides of 3′ untranslated region (utr) with the 3′ utr of the *abrupt* gene, which is transcribed in the opposite direction. Exon structure, direction of transcription (arrows), site of P-element insertion, and extent of the 5 Kb deletion in *cenp-metaΔ* mutation are indicated. (B) Western with Cenp-meta antibody shows one band above 250 kD in wild-type third instar larval brain protein extracts (WT) not found in larval brain protein extracts from *cenp-metaΔ* homozygous mutant (*cenp-metaΔ*). This band is consistent with the previously reported predicted size of Cenp-meta protein, which is 257 kD (Yucel et al., 2000). The same blot was stripped and reprobed with tubulin antibody to verify that equal amount of protein extracts were loaded for each lane. Asterisk indicates non-specific bands present in WT and *cenp-metaΔ* protein extracts.

Previous studies have shown that Cenp-E depletion leads to either a mitotic arrest in mammalian cells or a loss of mitotic arrest in *Xenopus* egg extracts. In order to address these apparent conflicting observations, we looked at the mitotic index in *cenp-metaΔ* mutant larval neuroblasts. The *cenp-metaΔ* allele is homozygous lethal at the larval stage (for further information on the nature of the *cenp-metaΔ* mutation, see Materials and Methods and Fig. 1A). No Cenp-meta protein was detected by western blot of *cenp-metaΔ* mutant protein extracts (Fig. 1B). Karyotypic analysis after staining chromosomes with aceto-orcein revealed a high level of aneuploidy, around 7% (Table 1; supplementary material Fig. S1). The functionality of the checkpoint in *cenp-metaΔ* mutant cells was tested by first looking if there was a mitotic arrest in the presence of colchicine, a microtubule depolymerizing agent. *cenp-metaΔ* mutant neuroblasts accumulated in M phase after 1 hour colchicine treatment, as the mitotic index increased by 2.46-fold, comparable to the 3-fold increase observed in wild type. Therefore, the checkpoint appeared to be functional in *cenp-metaΔ* mutant cells. To further confirm this, we generated a *cenp-metaΔ mad2^P^* double mutant and looked at the mitotic index in the presence of colchicine. *mad2^P^* is a null mutation that affects the spindle checkpoint but the cells display very little aneuploidy (Buffin et al., 2007). We found that the *cenp-metaΔ mad2^P^* double mutant flies were larval/pupal lethals, that the mitotic index no longer increased after treating the cells with colchicine (Table 1), and that the percentage of aneuploid cells was even higher than that observed in *cenp-metaΔ* single mutant (33.6% *vs* 6.7%). Therefore, these observations indicate that the spindle checkpoint appears to be functional in *cenp-metaΔ* mutant cells since removing the spindle checkpoint by removing Mad2 results in an even higher level of aneuploidy and lower mitotic index.

**Table 1.**
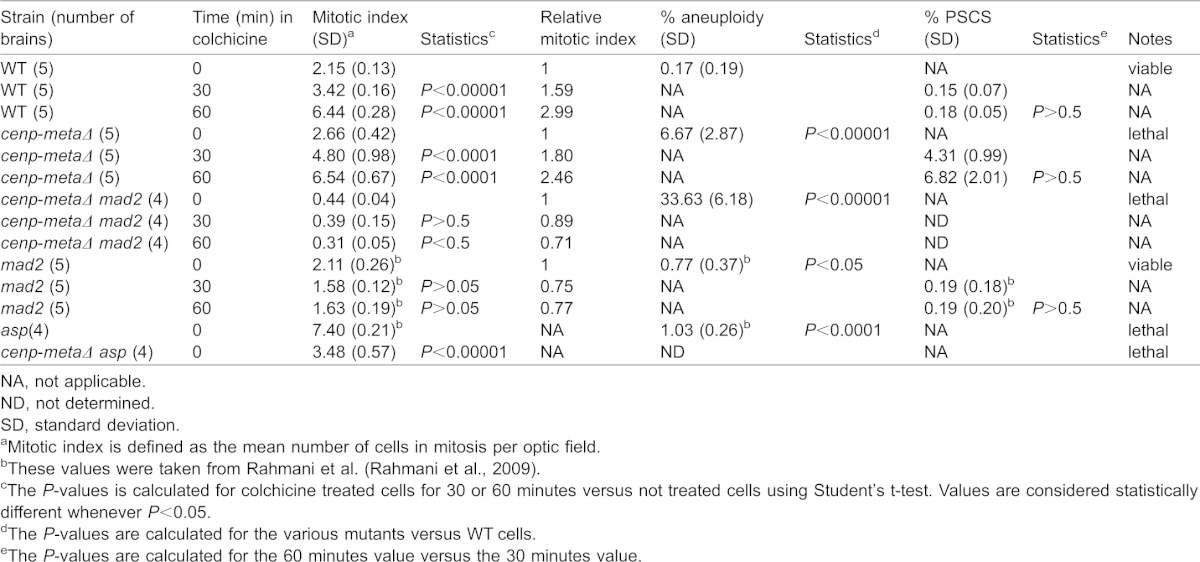
Analysis of the mitotic parameters in WT and *cenp-meta* mutant neuroblasts

**Fig. 2.**
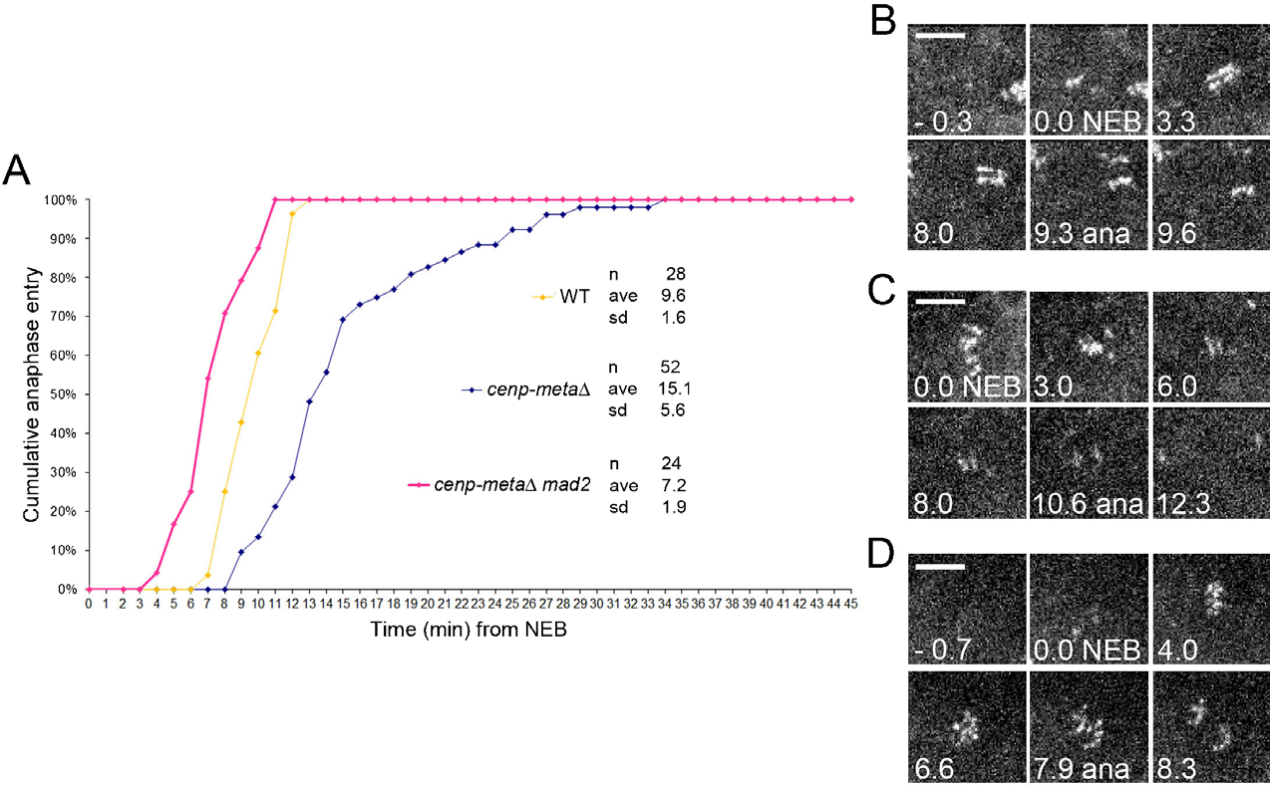
Mitotic timing in *cenp-metaΔ* mutant and *cenp-metaΔ mad2* double mutant neuroblasts. (A) Comparative mitotic timing of *cenp-metaΔ* and *cenp-metaΔ mad2* double mutant cells. NEB is defined as when RFP-Spc25 begins to be visible on kinetochores. *cenp-metaΔ* (blue diamonds) are delayed, with an average 15.1 minutes *vs* 9.6 minutes in WT (yellow diamonds). *cenp-metaΔ mad2* (pink diamonds) enter anaphase earlier than WT cells (7.2 minutes vs 9.6 minutes, *P*<0.005). *cenp-metaΔ mad2* double mutant cells (pink diamonds) show the same timing as the one that was previously reported for *mad2* alone (Buffin et al., 2007; Rahmani et al., 2009), thereby indicating that the prometaphase delay in *cenp-metaΔ* is SAC-dependent. (B–D) Still frames extracted from typical movies used for the determination of mitotic timing (from NEB to anaphase). (B) WT, (C) *cenp-metaΔ*, (D) *cenp-metaΔ mad2* double mutant. All cells are marked with RFP-Spc25. See also supplementary material Movies 1, 2, 3. Scale bars: 5 µm.

To further dissect the phenotype observed in the *cenp-metaΔ* single mutant, we analysed living *cenp-metaΔ* neuroblasts by time-lapse microscopy to determine the mitotic timing (i.e. the time that elapses between Nuclear Envelope Breakdown (NEB) and anaphase onset). For this, Spc25, a kinetochore protein that is part of the Ndc80 complex, tagged with the RFP fluorophore was expressed in *cenp-metaΔ* mutant cells to monitor the kinetochores. Whereas wild-type (wt) neuroblasts spent an average of 9.6±1.6 minutes (Fig. 2A,B; supplementary material Movie 1) between the NEB and the anaphase onset, *cenp-metaΔ* mutant neuroblasts showed a prolonged prometaphase with an average of 15.1±5.6 minutes (*P*<0.0001) (Fig. 2A,C; supplementary material Movie 2). We have shown previously that in *mad2* mutant cells, in which the spindle checkpoint is abolished, the average mitotic timing was accelerated (7.3 minutes) compared to wt cells (Buffin et al., 2007; Rahmani et al., 2009). To test if the spindle checkpoint was functional in *cenp-metaΔ* mutant cells, we measured the mitotic timing in *cenp-metaΔ mad2* double mutant cells and found that it was similarly accelerated (7.2±1.9 minutes, *P*<0.0001) (Fig. 2A,D; supplementary material Movie 3), thereby suggesting again that the spindle checkpoint is functioning in *cenp-metaΔ* mutant cells.

Paradoxically, despite the fact that the cells have an active checkpoint, the level of aneuploidy was very high in *cenp-metaΔ* single mutant cells (6.7% *vs* 0.2% in wt, Table 1). Moreover, the mutant cells treated with colchicine for 30 minutes or 60 minutes also displayed a high percentage of Premature Sister Chromatid Separation (PSCS) around 4.3% and 6.8%, respectively (Table 1; supplementary material Fig. S1). PSCS is generally considered a sign of mitotic exit and spindle checkpoint dysfunction.

To further explore this apparent weakness in the SAC function of *cenp-metaΔ*, we generated a double mutant of *cenp-metaΔ* and a null allele of *abnormal spindle* (*asp*). The *asp* mutation, which perturbs spindle assembly, normally causes cells to arrest for hours in mitosis (Ripoll et al., 1985) in a SAC-dependent manner (Basto et al., 2000; Buffin et al., 2007), and results in a very high mitotic index. We found that the mitotic index was significantly reduced in the *cenp-metaΔ asp* double mutant (3.5 *vs* 7.4 for *asp* alone, Table 1). This result confirms that the SAC is not as robust as in *asp* mutant cells. Therefore, the high level of aneuploid cells observed in *cenp-metaΔ* cells may be due to an overall reduction (but not elimination) of the SAC intensity or duration caused by the *cenp-metaΔ* mutation.

### The signal for the checkpoint proteins Mad2 and BubR1 persists on unaligned kinetochores present in *cenp-metaΔ* mutant cells

**Fig. 3.**
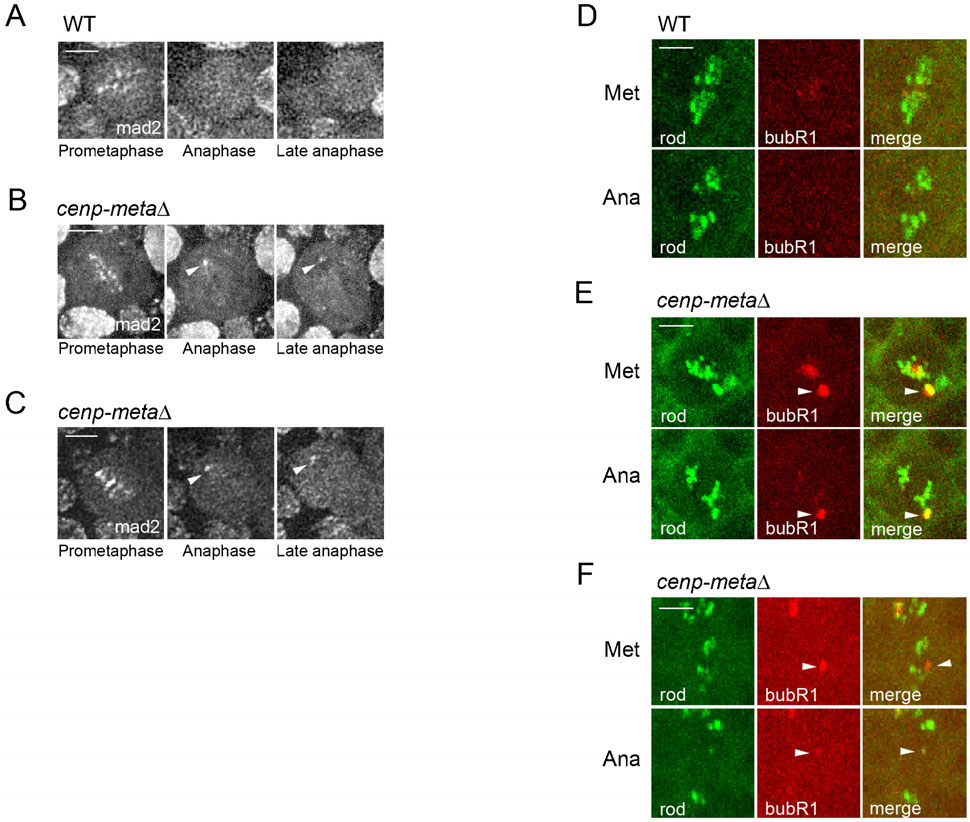
The checkpoint proteins Mad2 and BubR1 remain associated with the kinetochores of unaligned chromosomes in *cenp-metaΔ* mutant cells entering anaphase. (A–C) Selected frames from a WT (A) or two different *cenp-metaΔ* mutant cells (B,C) marked with GFP-Mad2 showing three mitotic stages (Prometaphase, Anaphase, Late anaphase). See the corresponding supplementary material Movies 4, 5, 6. Mad2 streaming during prometaphase is not affected in *cenp-metaΔ* mutant cells (B,C). While Mad2 signal is totally gone by the time the WT cell enters anaphase (A), it is still visible on kinetochores (arrowhead) of unaligned chromosomes at the poles and lasts until late anaphase in *cenp-metaΔ* mutant cells (B,C). (D–F) Selected frames from a WT (D) or two different *cenp-metaΔ* mutant cells (E,F) marked with GFP-Rod (green) and RFP-BubR1 (red). Note that GFP-Rod is used to monitor the anaphase onset since BubR1 signals are gone at this stage. BubR1 signal is no longer visible at metaphase (Met) in the WT cell (D) whereas it persists on unaligned kinetochores (arrowhead) seen in *cenp-metaΔ* mutant cells (E,F) entering anaphase (Ana). See also supplementary material Movies 7, 8, 9. Scale bars: 5 µm.

The possibility that the mitotic checkpoint may not be maintained in *cenp-metaΔ* mutant cells may be due to a reduced recruitment level of Mad2 and/or BubR1. In order to examine this, we looked at the dynamic behavior of these two checkpoint proteins during mitosis. For this, GFP-tagged Mad2 was expressed in wt or *cenp-metaΔ* mutant cells. As reported previously in wild-type neuroblasts, GFP-Mad2 was recruited to kinetochores of *cenp-metaΔ* mutant cells right at NEB (Buffin et al., 2005) and started very quickly to stream continuously toward the spindle poles during prometaphase. By the time, the cell reached anaphase, Mad2 signal was not visible anymore (Fig. 3A; supplementary material Movie 4). However, in *cenp-metaΔ* mutant cells, while the streaming of Mad2 appeared to be normal, Mad2 signal could still be detected on kinetochores of polar chromosomes by the time mutant cells underwent anaphase, and this signal lasted until late anaphase (Fig. 3B,C; supplementary material Movies 5, 6). The persistence of Mad2 on the unattached polar kinetochores is consistent with an active SAC in *cenp-metaΔ* mutant cells.

We similarly examined RFP-BubR1 in wt and *cenp-metaΔ* mutant cells. BubR1 signal at metaphase kinetochores was totally undetectable in wt cells (Fig. 3D; supplementary material Movie 7). However, strong BubR1 signals were detected on unaligned kinetochores observed in *cenp-metaΔ* mutant cells and stayed visible until late anaphase (Fig. 3E,F; supplementary material Movies 8, 9). Based on these observations, it appears that anaphase onset can still occur in *cenp-metaΔ* mutant cells even if Mad2 and BubR1 are still retained on unattached or misaligned kinetochores.

### Cyclin B is continuously degraded in *cenp-metaΔ* mutant cells

**Fig. 4.**
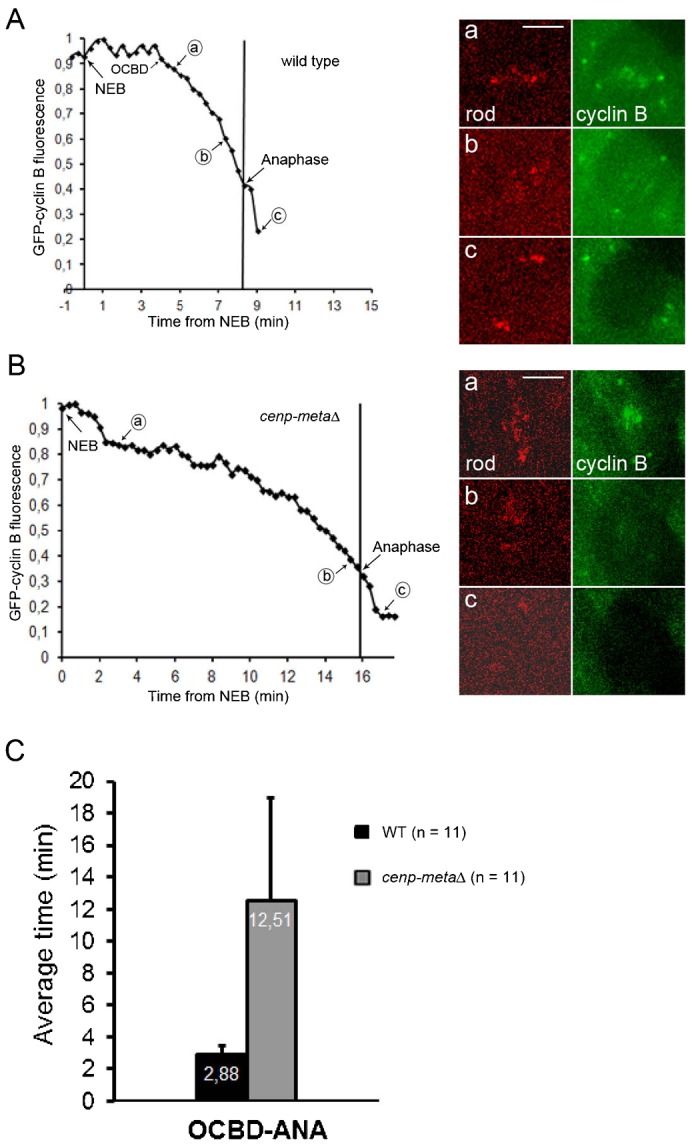
Cyclin B degradation profiles in WT and *cenp-metaΔ* mutant neuroblasts. Typical onset of cyclin B degradation (OCBD) in a single neuroblast. (A) WT, (B) *cenp-metaΔ* mutant neuroblast. (A,B) The frames showing RFP-Rod (red) and GFP-cyclin B (green) at various time points (indicated as a,b,c, on both graphs) in WT (A) and *cenp-metaΔ* mutant (B) cells were taken from supplementary material Movies 10 and 11, respectively. (C) Mean time of OCBD versus anaphase (ANA) onset. OCBD begins 2.9 minutes before anaphase onset in WT cells (see also Rahmani et al., 2009) whereas it is a slow continuous process in *cenp-metaΔ* mutant cells. See also supplementary material Movies 10, 11. Scale bars: 5 µm.

The level of inhibitory signal generated by Mad2 and BubR1 on unattached kinetochores observed in *cenp-metaΔ* mutant cells may not be high enough over time to efficiently suppress APC/C activation and consequently APC/C-mediated degradation of mitotic substrates such as cyclin B that leads to inactivation of Cdk1 activity and mitotic exit. Thus, measurement of cyclin B degradation during mitosis in wt and *cenp-metaΔ* mutant cells was used as an *in vivo* readout of APC/C activation. In agreement with what we showed previously (Buffin et al., 2007; Rahmani et al., 2009), the onset on cyclin B degradation (OCBD) in wt *Drosophila* neuroblasts expressing GFP-tagged cyclin B started between 4 to 7 minutes after NEB and the timing between OCBD and anaphase onset was relatively constant with an average of 2.9±0.6 minutes (Fig. 4A,C; supplementary material Movie 10). However, in *cenp-metaΔ* mutant cells, the levels of cyclin B declined gradually but constantly until the cell entered anaphase (Fig. 4B,C; supplementary material Movie 11). This result suggests that in *cenp-metaΔ* mutant cells, the functional SAC cannot effectively block APC/C mediated cyclin B degradation. Consequently, the continuous degradation of cyclin B mediated by the activated APC/C could eventually reach a threshold for which mitotic arrest could no longer be maintained.

## DISCUSSION

Here we have provided several lines of evidence that Cenp-meta is required for long-term maintenance of the spindle checkpoint. First, while *cenp-metaΔ* mutant cells arrested in mitosis when treated with colchicine (as reflected by the increased number of mitotic cells seen in *cenp-metaΔ* mutant treated cells), the relatively high level of cells with PSCS revealed that these cells were not able to maintain a prolonged arrest when treated with spindle poisons. Second, the high mitotic index caused by the *asp* mutation is strongly reduced when combined with *cenp-metaΔ* mutation, thereby suggesting that somehow Cenp-meta helps to maintain the SAC intensity or duration. Third, the persistence of the checkpoint components BubR1 and Mad2 on kinetochores of unaligned chromosomes did not preclude *cenp-metaΔ* mutant cells from entering anaphase. Fourth, in WT cells, cyclin B levels start to decline rapidly around 2–3 minutes before anaphase onset, whereas, in *cenp-metaΔ* mutant cells, cyclin B degradation is a slow but continuous process.

Two very recent reports showed that the strength of the SAC response is graded and depends not only on the number of unattached kinetochores but also on the kinetochore levels of Mad2 (Collin et al., 2013; Dick and Gerlich, 2013). Although this may contribute to explain why untreated *cenp-metaΔ* mutant cells, with only a few mono-oriented kinetochores, may still enter anaphase and produce aneuploid cells, it does not adequately explain some other aspects of the *cenp-metaΔ* phenotype. Indeed, in colchicine-treated cells, despite the fact that all kinetochores are unattached and so generate a SAC signal, the SAC appears to be less robust in the *cenp-metaΔ* mutant than in the wt as revealed by the observation of a high percentage of *cenp-metaΔ* mutant cells with PSCS. Moreover, the reduction of the high mitotic index observed in the *cenp-metaΔ asp* double mutant provides compelling evidence that Cenp-meta itself may contribute to the SAC response. Therefore, an alternate explanation is that *cenp-metaΔ* may attenuate SAC signal directly by interfering with its production or its maintenance. Cenp-meta may possibly influence BubR1 kinase activity. In *Xenopus* mitotic egg extracts and primary mouse fibroblasts, Cenp-E was shown to form a ternary complex that was necessary for the activation of BubR1 kinase activity (Abrieu et al., 2000; Mao et al., 2003; Mao et al., 2005; Weaver et al., 2003). More recently, Guo et al. demonstrated that kinetochore-associated BubR1 phosphorylates itself on T608 in human cells *in vivo* and that this phosphorylation is dependent on kinetochore-associated Cenp-E (Guo et al., 2012). However, Suijkerbuijk et al. reported that most vertebrate BubR1 (but not *Drosophila* BubR1) was more likely to be a pseudokinase with no catalytic activity and that the pseudokinase domain was important for BubR1 protein stability (Suijkerbuijk et al., 2012). Thus, whether vertebrate BubR1 possesses real kinase activity still remains a controversial issue. However, the three catalytic residues essential for a conventional kinase are retained in *Drosophila* BubR1 domain (Suijkerbuijk et al., 2012), and suggest that *Drosophila* BubR1 may potentially be catalytically active. If it is the case, the absence of Cenp-meta may affect *Drosophila* BubR1 potential kinase activity in *cenp-metaΔ* mutant cells, thereby mimicking a phenotype that resembles the one observed with BubR1-KD (Kinase mutated) expressing cells. We showed previously that the potential kinase activity of BubR1 is dispensable for initiating the spindle checkpoint response in *Drosophila* larval neuroblasts (Rahmani et al., 2009) but the presence of PSCS in BubR1-KD expressing cells treated with colchicine for 60 minutes suggests that the potential BubR1 kinase activity may be needed for long-term maintenance of metaphase arrest (Rahmani et al., 2009). Interestingly, the percentage of cells with PSCS in *cenp-metaΔ* mutant is much higher when compared to *bubR1-KD* mutant (6.8±2 *vs* 1.4±0.7, Table 1; Rahmani et al., 2009; respectively). This indicates that the percentage of cells that display signs of premature mitotic exit (reflected by the presence of PSCS) after colchicine treatment is greater in the absence of Cenp-meta than in the absence of BubR1 potential kinase activity and suggests that Cenp-meta may also affect the SAC independently of its presumed function in modulating BubR1 potential kinase activity. In conclusion, we have shown that Cenp-meta is important for prolonged SAC-dependent mitotic arrest. Whether Cenp-meta acts through BubR1 potential kinase activity and/or an unknown mechanism remains to be tested.

## Supplementary Material

Supplementary Material
