# Supplementary material for: Cenp-meta is required for sustained spindle checkpoint
Source: Biol Open. 2014 May 29;3(6):522–8. doi: 10.1242/bio.20148490 (PMC4058087; doi:10.1242/bio.20148490)
Supplement: Supplementary Material [file supp_3_6_522__index.html]

Cenp-meta is required for sustained spindle checkpoint — Cenp-meta is required for sustained spindle checkpoint — Supplementary Material 

# Cenp-meta is required for sustained spindle checkpoint

## bio.20148490 Supplementary Material

**Files in this Data Supplement:**

- Supplementary Material - Thomas Rubin et al. doi: 10.1242/bio.20148490
- Movie 1 - **Movie 1. Mitotic timing in a WT neuroblast.** WT *Drosophila* larval neuroblast labeled with RFP-Spc25 (red). Corresponds to Fig. 2B. Images were acquired by confocal spinning disk microscopy. Frames were taken every 20 seconds for 9.6 minutes. The movie is shown at 8 frames/s.
- Movie 2 - **Movie 2. Mitotic timing in a *cenp-metaΔ* mutant neuroblast.** *cenp-metaΔ* mutant *Drosophila* larval neuroblast labeled with RFP-Spc25 (red). Corresponds to Fig. 2C. Images were acquired by confocal spinning disk microscopy. Frames were taken every 20 seconds for 13.6 minutes. The movie is shown at 8 frames/s.
- Movie 3 - **Movie 3. Mitotic timing in a *cenp-metaΔ mad2P* mutant neuroblast.** *cenp-metaΔ mad2P* mutant *Drosophila* larval neuroblast labeled with RFP-Spc25 (red). Corresponds to Fig. 2D. Images were acquired by confocal spinning disk microscopy. Frames were taken every 20 seconds for 9.3 minutes. The movie is shown at 8 frames/s.
- Movie 4 - **Movie 4. Mad2 dynamics in a WT neuroblast.** WT *Drosophila* larval neuroblast labeled with GFP-Mad2 (green). Corresponds to Fig. 3A. Images were acquired by confocal spinning disk microscopy. Frames were taken every 20 seconds for 16.3 minutes. The movie is shown at 8 frames/s.
- Movie 5 - **Movie 5. Mad2 dynamics in a *cenp-metaΔ* mutant neuroblast.** *cenp-metaΔ* mutant *Drosophila* larval neuroblast labeled with GFP-Mad2 (green). Corresponds to Fig. 3B. Images were acquired by confocal spinning disk microscopy. Frames were taken every 20 seconds for 10.7 minutes. The movie is shown at 8 frames/s.
- Movie 6 - **Movie 6. Mad2 dynamics in a *cenp-metaΔ* mutant neuroblast.** *cenp-metaΔ* mutant *Drosophila* larval neuroblast labeled with GFP-Mad2 (green). Corresponds to Fig. 3C. Images were acquired by confocal spinning disk microscopy. Frames were taken every 20 seconds for 14.7 minutes. The movie is shown at 8 frames/s.
- Movie 7 - **Movie 7. BubR1 dynamics in a WT neuroblast.** WT *Drosophila* larval neuroblast labeled with GFP-rod (green; left) and RFP-BubR1 (red; middle). Merged images are shown in the right panel. Corresponds to Fig. 3D. Images were acquired by confocal spinning disk microscopy. Frames were taken every 20 seconds for 5 minutes. The movie is shown at 8 frames/s.
- Movie 8 - **Movie 8. BubR1 dynamics in a *cenp-metaΔ* mutant neuroblast.** *cenp-metaΔ* mutant *Drosophila* larval neuroblast labeled with GFP-rod (green; left) and RFP-BubR1 (red; middle). Merged images are shown in the right panel. Corresponds to Fig. 3E. Images were acquired by confocal spinning disk microscopy. Frames were taken every 20 seconds for 8.3 minutes. The movie is shown at 8 frames/s.
- Movie 9 - **Movie 9. BubR1 dynamics in a *cenp-metaΔ* mutant neuroblast.** *cenp-metaΔ* mutant *Drosophila* larval neuroblast labeled with GFP-rod (green; left) and RFP-BubR1 (red; middle). Merged images are shown in the right panel. Corresponds to Fig. 3F. Images were acquired by confocal spinning disk microscopy. Frames were taken every 20 seconds for 4.3 minutes. The movie is shown at 8 frames/s.
- Movie 10 - **Movie 10. Cyclin B degradation in a WT neuroblast.** WT *Drosophila* larval neuroblast labeled with GFP-cyclin B (green; top left) and RFP-Rod (red; top right). Merged images are shown in the bottom left panel. Corresponds to Fig. 4A. Images were acquired by confocal spinning disk microscopy. Frames were taken every 20 seconds for 8.9 minutes. The movie is shown at 8 frames/s.
- Movie 11 - **Movie 11. Cyclin B degradation in a *cenp-metaΔ* mutant neuroblast.** *cenp-metaΔ* mutant *Drosophila* larval neuroblast labeled with GFP-cyclin B (green; top left) and RFP-Rod (red; top right). Merged images are shown in the bottom left panel. Corresponds to Fig. 4B. Images were acquired by confocal spinning disk microscopy. Frames were taken every 20 seconds for 17.2 minutes. The movie is shown at 8 frames/s.
