# Supplementary material for: Cenp-meta is required for sustained spindle checkpoint
Source: Biol Open. 2014 May 29;3(6):522–8. doi: 10.1242/bio.20148490 (PMC4058087; doi:10.1242/bio.20148490)
Supplement: Supplementary Material [file supp_bio.20148490_bio.20148490-s1.pdf]

Biology Open (2014) 000, 1–7 doi:10.1242/bio.20148490

Supplementary Material

Thomas Rubin et al. doi: 10.1242/bio.20148490

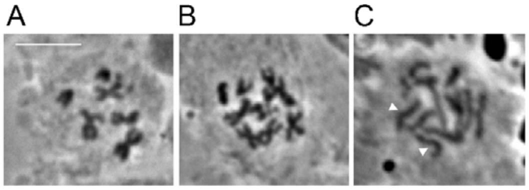

Fig. S1. Mitotic defects in the *cenp-metaΔ* mutant. Wild-type (A) and *cenp-metaΔ* mutant (B) brains were fixed in acetic acid and then squashed in aceto-orcein. (A) Wild-type prometaphase. (B) Aneuploid cell in *cenp-metaΔ* mutant. (C) Note the presence of PSCS (arrowheads) in *cenp-metaΔ* mutant cell treated with colchicine for 60 minutes. Scale bar: 5 µm.

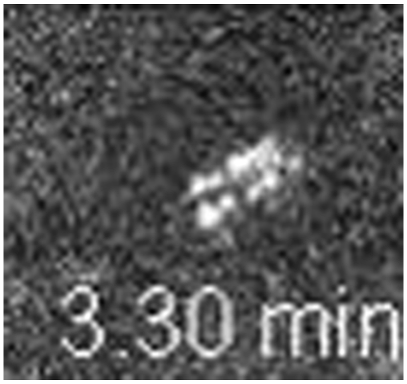

Movie 1. Mitotic timing in a WT neuroblast. WT *Drosophila* larval neuroblast labeled with RFP-Spc25 (red). Corresponds to Fig. 2B. Images were acquired by confocal spinning disk microscopy. Frames were taken every 20 seconds for 9.6 minutes. The movie is shown at 8 frames/s.

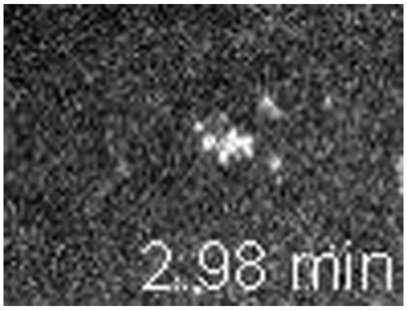

Movie 2. Mitotic timing in a *cenp-metaΔ* mutant neuroblast. *cenp-metaΔ* mutant *Drosophila* larval neuroblast labeled with RFP-Spc25 (red). Corresponds to Fig. 2C. Images were acquired by confocal spinning disk microscopy. Frames were taken every 20 seconds for 13.6 minutes. The movie is shown at 8 frames/s.

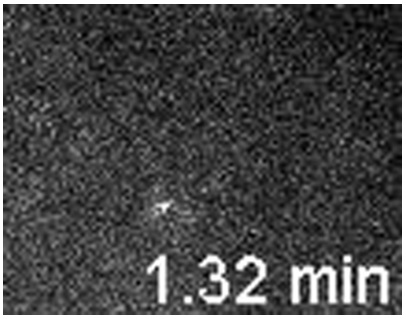

Movie 3. Mitotic timing in a *cenp-metaΔ mad2^P* mutant neuroblast. *cenp-metaΔ mad2^P* mutant *Drosophila* larval neuroblast labeled with RFP-Spc25 (red). Corresponds to Fig. 2D. Images were acquired by confocal spinning disk microscopy. Frames were taken every 20 seconds for 9.3 minutes. The movie is shown at 8 frames/s.

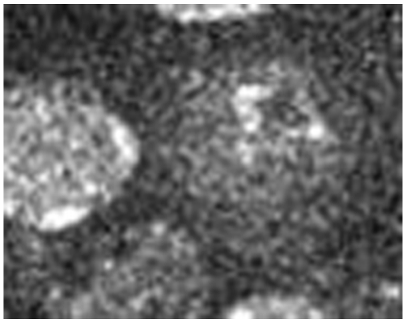

Movie 4. Mad2 dynamics in a WT neuroblast. WT *Drosophila* larval neuroblast labeled with GFP-Mad2 (green). Corresponds to Fig. 3A. Images were acquired by confocal spinning disk microscopy. Frames were taken every 20 seconds for 16.3 minutes. The movie is shown at 8 frames/s.

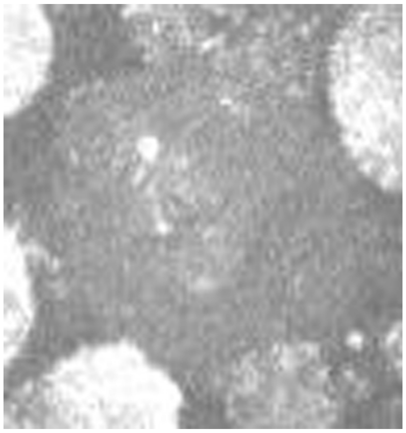

Movie 5. Mad2 dynamics in a *cenp-metaΔ* mutant neuroblast. *cenp-metaΔ* mutant *Drosophila* larval neuroblast labeled with GFP-Mad2 (green). Corresponds to Fig. 3B. Images were acquired by confocal spinning disk microscopy. Frames were taken every 20 seconds for 10.7 minutes. The movie is shown at 8 frames/s.

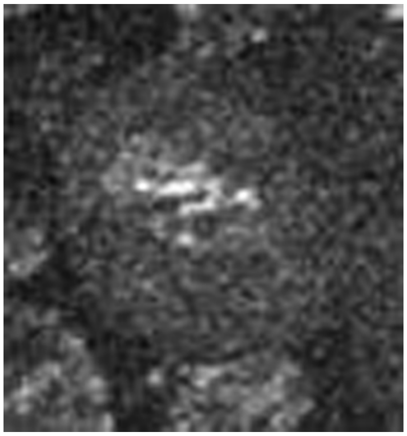

Movie 6. Mad2 dynamics in a *cenp-metaΔ* mutant neuroblast. *cenp-metaΔ* mutant *Drosophila* larval neuroblast labeled with GFP-Mad2 (green). Corresponds to Fig. 3C. Images were acquired by confocal spinning disk microscopy. Frames were taken every 20 seconds for 14.7 minutes. The movie is shown at 8 frames/s.

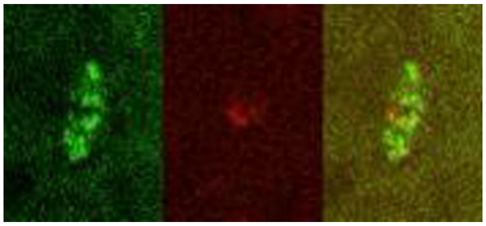

Movie 7. BubR1 dynamics in a WT neuroblast. WT *Drosophila* larval neuroblast labeled with GFP-rod (green; left) and RFP-BubR1 (red; middle). Merged images are shown in the right panel. Corresponds to Fig. 3D. Images were acquired by confocal spinning disk microscopy. Frames were taken every 20 seconds for 5 minutes. The movie is shown at 8 frames/s.

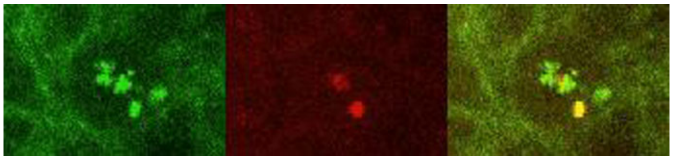

Movie 8. BubR1 dynamics in a *cenp-metaΔ* mutant neuroblast. *cenp-metaΔ* mutant *Drosophila* larval neuroblast labeled with GFP-rod (green; left) and RFP-BubR1 (red; middle). Merged images are shown in the right panel. Corresponds to Fig. 3E. Images were acquired by confocal spinning disk microscopy. Frames were taken every 20 seconds for 8.3 minutes. The movie is shown at 8 frames/s.

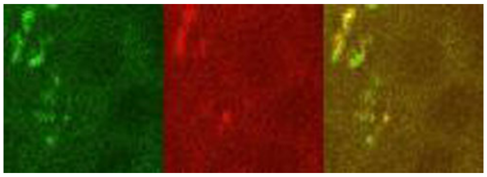

Movie 9. BubR1 dynamics in a *cenp-metaΔ* mutant neuroblast. *cenp-metaΔ* mutant *Drosophila* larval neuroblast labeled with GFP-rod (green; left) and RFP-BubR1 (red; middle). Merged images are shown in the right panel. Corresponds to Fig. 3F. Images were acquired by confocal spinning disk microscopy. Frames were taken every 20 seconds for 4.3 minutes. The movie is shown at 8 frames/s.

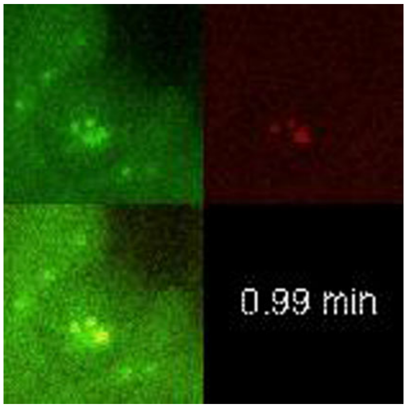

Movie 10. Cyclin B degradation in a WT neuroblast. WT *Drosophila* larval neuroblast labeled with GFP-cyclin B (green; top left) and RFP-Rod (red; top right). Merged images are shown in the bottom left panel. Corresponds to Fig. 4A. Images were acquired by confocal spinning disk microscopy. Frames were taken every 20 seconds for 8.9 minutes. The movie is shown at 8 frames/s.

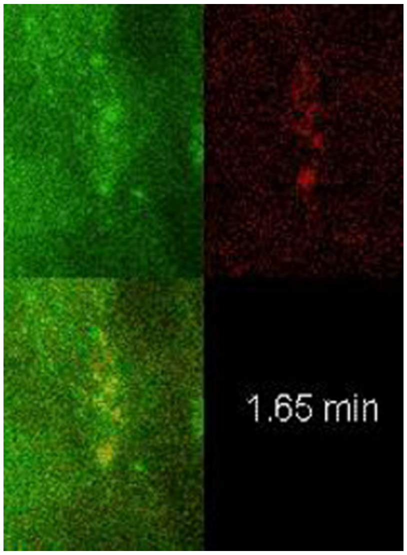

Movie 11. Cyclin B degradation in a *cenp-metaΔ* mutant neuroblast. *cenp-metaΔ* mutant *Drosophila* larval neuroblast labeled with GFP-cyclin B (green; top left) and RFP-Rod (red; top right). Merged images are shown in the bottom left panel. Corresponds to Fig. 4B. Images were acquired by confocal spinning disk microscopy. Frames were taken every 20 seconds for 17.2 minutes. The movie is shown at 8 frames/s.
